# Transmembrane serine protease TMPRSS2 implicated in SARS-CoV-2 infection is autoactivated intracellularly and requires N-glycosylation for regulation

**DOI:** 10.1016/j.jbc.2022.102643

**Published:** 2022-10-26

**Authors:** Yikai Zhang, Shijin Sun, Chunyu Du, Kaixuan Hu, Ce Zhang, Meng Liu, Qingyu Wu, Ningzheng Dong

**Affiliations:** 1 Cyrus Tang Hematology Center, Collaborative Innovation Center of Hematology, State Key Laboratory of Radiation Medicine and Prevention, Suzhou Medical College, Soochow University, Suzhou, China; 2 NHC Key Laboratory of Thrombosis and Hemostasis, Jiangsu Institute of Hematology, The First Affiliated Hospital of Soochow University, Suzhou, China

**Keywords:** HAI-1, HAI-2, N-glycosylation, SARS-CoV-2, spike protein, TMPRSS2, TTSP, zymogen activation, BFA, brefeldin A, BiP, binding immunoglobulin protein, cDNA, complementary DNA, CoV, coronavirus, DNJ, 1-deoxynojirimycin, ER, endoplasmic reticulum, FBS, fetal bovine serum, HAI, hepatocyte growth factor activator inhibitor, HEK293, human embryonic kidney 293 cell line, HRP, horseradish peroxidase, HSP70, heat shock protein 70, LDLR, low-density lipoprotein receptor, MEM, minimal essential medium, PNGase F, peptide-*N*-glycosidase F, S, spike protein, SARS, severe acute respiratory syndrome, SRCR, scavenger receptor cysteine-rich domain, STR, short tandem repeat, TMPRSS2, transmembrane protease serine 2, TTSP, type II transmembrane serine protease

## Abstract

Transmembrane protease serine 2 (TMPRSS2) is a membrane-bound protease expressed in many human epithelial tissues, including the airway and lung. TMPRSS2-mediated cleavage of viral spike protein is a key mechanism in severe acute respiratory syndrome coronavirus 2 activation and host cell entry. To date, the cellular mechanisms that regulate TMPRSS2 activity and cell surface expression are not fully characterized. In this study, we examined two major post-translational events, zymogen activation and N-glycosylation, in human TMPRSS2. In experiments with human embryonic kidney 293, bronchial epithelial 16HBE, and lung alveolar epithelial A549 cells, we found that TMPRSS2 was activated *via* intracellular autocatalysis and that this process was blocked in the presence of hepatocyte growth factor activator inhibitors 1 and 2. By glycosidase digestion and site-directed mutagenesis, we showed that human TMPRSS2 was N-glycosylated. N-glycosylation at an evolutionarily conserved site in the scavenger receptor cysteine-rich domain was required for calnexin-assisted protein folding in the endoplasmic reticulum and subsequent intracellular trafficking, zymogen activation, and cell surface expression. Moreover, we showed that TMPRSS2 cleaved severe acute respiratory syndrome coronavirus 2 spike protein intracellularly in human embryonic kidney 293 cells. These results provide new insights into the cellular mechanism in regulating TMPRSS2 biosynthesis and function. Our findings should help to understand the role of TMPRSS2 in major respiratory viral diseases.

Transmembrane protease serine 2 (TMPRSS2) is a member of the type II transmembrane serine protease (TTSP) family (1, 2, 3), which includes hepsin, matriptase, human airway trypsin-like protease, and corin that are of physiological importance (4, 5, 6, 7, 8, 9, 10, 11). TMPRSS2 is expressed in many epithelial tissues, including the prostate, trachea, bronchus, lung, kidney, small intestine, colon, pancreas, thymus, and salivary glands (12, 13, 14). To date, the physiological function of TMPRSS2 remains elusive. In mice, disruption of the *Tmprss2* gene does not cause noticeable changes in embryonic development, postnatal survival, fertility, prostate morphology, and kidney function (15). In humans, *TMPRSS2* overexpression and gene rearrangements have been identified as an underlying mechanism in prostate cancer development and progression (16, 17, 18).

TTSPs are anchored on the cell surface *via* an N-terminal transmembrane domain (2, 3). In human airways, TTSPs are exploited by coronaviruses (CoVs), including the severe acute respiratory syndrome (SARS) CoV, the Middle East respiratory syndrome CoV, and SARS-CoV-2, for their infectivity. In these CoVs, spike (S) proteins on the viral envelope are responsible for cellular receptor binding, membrane fusion, and cell entry (19, 20, 21). Proteolytic cleavage of S proteins by host TTSPs, including human airway trypsin-like protease, TMPRSS2, TMPRSS4, TMPRSS11A, and TMPRSS11E, enhances S protein activity and CoV infection (22, 23, 24, 25, 26, 27).

To date, ample evidence indicates that TMPRSS2-mediated S protein cleavage is a critical mechanism in SARS-CoV-2 activation and cell entry. In cultured human lung epithelial cells, TMPRSS2 expression enhances SARS-CoV-2 infection, whereas inhibition of TMPRSS2 expression or activity blocks the viral entry (28, 29, 30, 31). In a mouse *Tmprss2* knockout model, SARS-CoV-2 infection in the lung is markedly reduced compared with that in WT mice (32). Similarly, a small-molecule TMPRSS2 inhibitor is shown to provide prophylactic and therapeutic benefits in a mouse model of severe coronavirus disease 2019 (33). In addition to SARS-CoV-2, TMPRSS2 also enhances the activity of SARS and Middle East respiratory syndrome CoVs and influenza A viruses by cleaving S proteins and hemagglutinins, respectively (13, 22, 34, 35, 36). These data underscore a key role of TMPRSS2 in major viral respiratory diseases.

Most TTSPs are synthesized in a single-chain zymogen form. Post-translational modifications, including N- or O-glycosylation, zymogen activation, phosphorylation, and ectodomain cleavage, are important mechanisms in regulating TTSP activities (37, 38, 39, 40, 41, 42, 43, 44, 45). Previous studies indicate that TMPRSS2 is synthesized as a single-chain zymogen and activated by autocatalysis (46). To date, the cellular mechanism that regulates TMPRSS2 activation and cell surface expression is not fully understood. In this study, we examined TMPRSS2 zymogen activation and N-glycosylation in biochemical and cellular experiments. Our results show that TMPRSS2 is activated intracellularly by autocatalysis in human kidney, airway, and lung-derived cells and that N-glycosylation in the scavenger receptor cysteine-rich (SRCR) domain is critical for TMPRSS2 activation and cell surface expression. We also show that TMPRSS2 cleaves SARS-CoV-2 S protein intracellularly. These findings help to understand the cellular mechanism underlying TMPRSS2 activation and function.

## Results

### TMPRSS2 autoactivation in human cells

**Figure 1:**
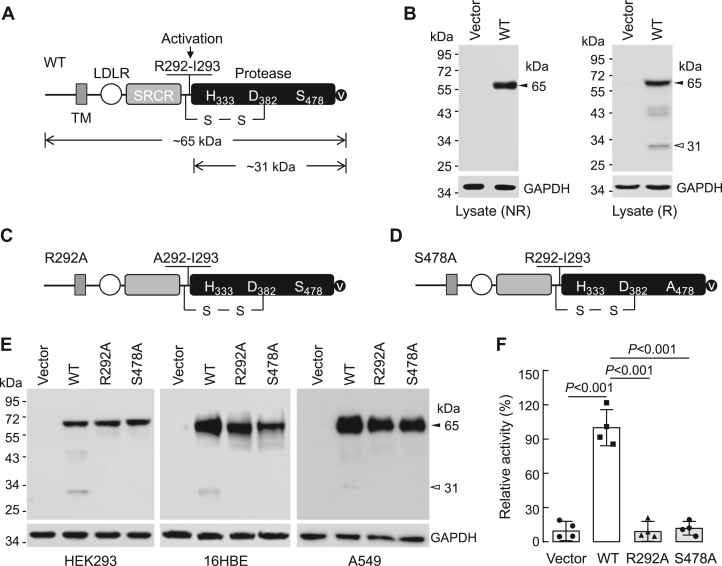
**TMPRSS2 autoactivation in human cells.***A*, illustration of human TMPRSS2 domains. LDLR, low-density lipoprotein receptor domain; SRCR, scavenger receptor cysteine-rich domain; TM, transmembrane domain. The zymogen activation site, catalytic active residues (H333, D382, and S478) in the protease domain, and a disulfide bond between the propeptide and protease domains are indicated. A V5 (*v*) tag is at the C terminus. Predicted sizes of TMPRSS2 zymogen and the protease domain are indicated. *B*, Western blotting of TMPRSS2 in lysates from HEK293 cells transfected with a vector or a plasmid expressing TMPRSS2 WT under nonreducing (*NR*) (*left*) or reducing (*R*) (*right*) conditions. The ∼65- and ∼31-kDa bands are indicated by *filled* and *open arrowheads*, respectively. GAPDH was a control. *C* and *D*, illustrations of TMPRSS2 mutants R292A (*C*) and S478A (*D*). *E*, Western blotting of the TMPRSS2 WT and mutants R292A and S478A in lysates from transfected HEK293, 16HBE, and A549 cells under reducing conditions. *F*, peptide substrate activities in HEK293 cells transfected with a vector or plasmids expressing the WT and mutants R292A and S478A (n = 4). Data in *B* and *E* are representative of at least three experiments. Data in *F* are mean ± SD, analyzed by one-way ANOVA. HEK293, human embryonic kidney 293 cell line; TMPRSS2, transmembrane protease serine 2.

Figure 1*A* is an illustration of human TMPRSS2 protein domains, including an N-terminal cytoplasmic tail, a transmembrane domain, a low-density lipoprotein receptor (LDLR)–like domain, an SRCR domain, and a C-terminal trypsin-like protease domain with three canonical active sites, His333 (H333), Asp382 (D382), and Ser478 (S478). The conserved zymogen activation site is at R292–I293. There is a disulfide bond between the propeptide and the protease domain (Fig. 1*A*). To study TMPRSS2, we transfected human embryonic kidney 293 (HEK293) cells with a plasmid expressing human TMPRSS2 with a C-terminal V5 tag (Fig. 1*A*). In Western blotting of lysates from the transfected cells, we detected a single band of ∼65 kDa under nonreducing conditions (Figs. 1*B*, *left panel* and S1*A*). Under reducing conditions, we detected an ∼65-kDa band and an ∼31-kDa band (Figs. 1*B*, *right panel* and S1*A*), representing the zymogen and the cleaved protease domain fragment, respectively (Fig. 1*A*). Because the V5 tag was at the C terminus, the cleaved N-terminal fragment was not detected. There were two additional lighter bands of ∼45 to 48 kDa, probably generated by cleavages between the LDLR and the SRCR domains.

To understand how the TMPRSS2 fragments were generated, we made TMPRSS2 R292A and S478A mutants, in which the canonical activation site (Fig. 1*C*) and the catalytic serine (Fig. 1*D*) were mutated, respectively. Both the mutants are expected to be catalytically inactive. We expressed TMPRSS2 WT and mutants R292A and S478A in HEK293 cells. In Western blotting under reducing conditions, we detected the ∼65-kDa, but not the ∼31-kDa, band in the mutants R292A and S478A (Figs. 1*E*, *left panel* and S1*B*). Similar results were found when the WT and mutants were expressed in human bronchial epithelial 16HBE cells (Figs. 1*E*, *middle panel* and S1*B*) and human alveolar basal epithelial A549 cells (Figs. 1*E*, *right panel* and S1*B*). These results indicated that the ∼31-kDa band is the protease domain cleaved at R292 and that the cleavage depends on the catalytic activity of TMPRSS2.

To verify these results, we examined the catalytic activity of TMPRSS2 expressed in HEK293 cells. In a fluorogenic substrate assay, significant catalytic activity was detected in the cells expressing the TMPRSS2 WT, whereas little activity was found in the vector-transfected cells or the cells expressing the mutants R292A and S478A (Fig. 1*F*). These results are consistent, indicating that the TMPRSS2 WT is activated in HEK293 cells.

### TMPRSS2 expression on the cell surface

Type II transmembrane protein, TMPRSS2, is expected to be on the cell surface. To verify its cell surface location, we biotin labeled membrane proteins in HEK293 cells and analyzed TMPRSS2 proteins by Western blotting. Surprisingly, we detected the mutants R292A and S478A, but not the WT, in biotin-labeled protein fractions (Fig. S2*A*, *left panel*). In cell lysates, levels of the WT and mutants were comparable (Fig. S2*A*, *right panel*). We confirmed these results by flow cytometry, which showed the mutants, but not the WT, on the HEK293 cell surface (Fig. S2*B*). Moreover, we observed immunostaining of the mutants, but not the WT, in nonpermeabilized HEK293 cells, whereas immunostaining of the WT and mutants was observed in permeabilized HEK293 cells (Fig. S2*C*). Considering that the aforementioned experiments were done using an anti-C-terminal V5 tag antibody, it is possible that the activated TMPRSS2 WT cleaved the V5 tag, preventing the antibody detection, whereas in the inactive mutants, R292A and S478A, the V5 tag was not cleaved and thus detectable.

**Figure 2:**
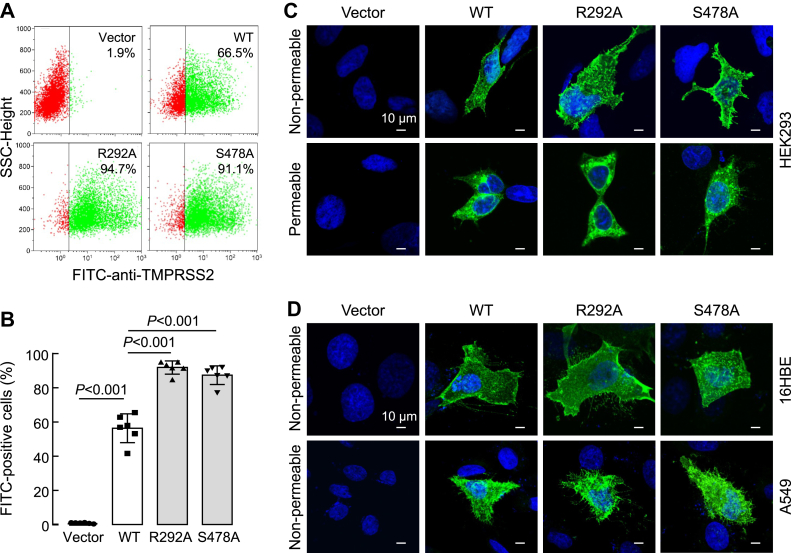
**Analysis of cell surface TMPRSS2 proteins.***A* and *B*, flow cytometric analysis of TMPRSS2 proteins on the surface of HEK293 cells transfected with a vector or plasmids expressing the TMPRSS2 WT and mutants R292A and S478A. Percentages of TMPRSS2-positive cells are indicated (*A*). Quantitative data of FITC-positive cells (mean ± SD) from six experiments were analyzed by one-way ANOVA (*B*). *C* and *D*, immunostaining of TMPRSS2 proteins (*green*) in HEK293 cells (*C*) and human airway 16HBE and lung A549 cells (*D*) transfected with a vector or plasmids expressing the WT and mutants R292A and S478A. Cell nuclei were stained with DAPI (*blue*). Experiments were done under cell membrane nonpermeabilized or permeabilized conditions, as indicated. Both flow cytometry and immunostaining were done using an antibody against an epitope in the TMPRSS2 extracellular stem region. In *C* and *D*, data are representative of at least three experiments. The scale bars represent 10 μm. DAPI, 4′,6-diamidino-2-phenylindole; HEK293, human embryonic kidney 293 cell line; TMPRSS2, transmembrane protease serine 2.

To verify this hypothesis, we tested another antibody against an extracellular stem region of human TMPRSS2. (The specific epitope was undisclosed by the commercial maker.) In flow cytometry, the antibody detected the TMPRSS2 WT and mutants R292A and S478A on the HEK293 cell surface (Fig. 2*A*). Levels of the cell surface WT were lower than those of the mutants (Fig. 2*B*), probably because of ectodomain shedding of the WT. In immunostaining, the WT and mutants were detected on the surface of nonpermeabilized and within permeabilized HEK293 cells (Fig. 2*C*). Similarly, the cell surface expression of the WT and mutants was detected in human airway 16HBE and lung A549 cells by immunostaining (Fig. 2*D*) and flow cytometry (Fig. S3, *A* and *B*).

### Intracellular activation of TMPRSS2

**Figure 3:**
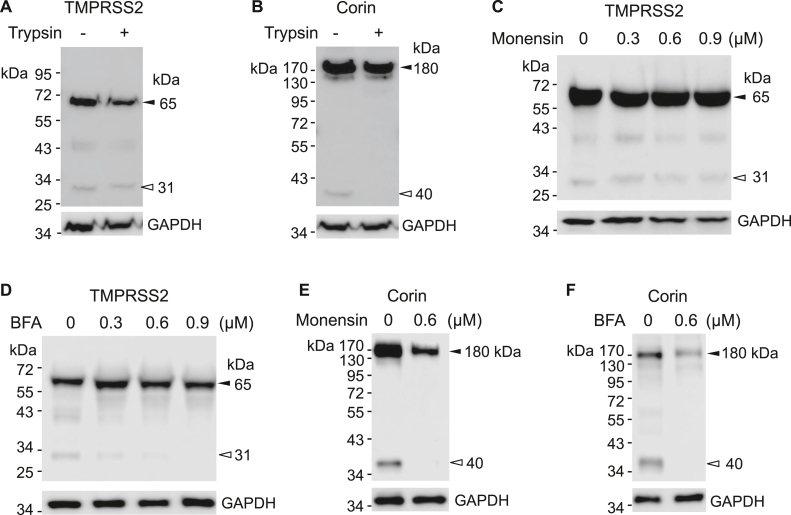
**Analysis of TMPRSS2 activation cleavage in trypsin-, monensin-, or BFA-treated HEK293 cells.***A* and *B*, Western blotting of TMPRSS2 (*A*) and corin (*B*) in lysates from transfected HEK293 without (−) or with (*+*) trypsin pretreatment. The blotting was done under reducing conditions. Activation-cleaved protease domain bands from TMPRSS2 (∼31 kDa in *A*) and corin (∼40 kDa in *B*) are indicated by *open arrowheads*. GAPDH was shown as a control. *C* and *D*, HEK293 cells expressing TMPRSS2 were treated with increasing doses of monensin (*C*) or BFA (*D*) at 37 °C for 18 h. TMPRSS2 in cell lysates was analyzed by Western blotting under reducing conditions. TMPRSS2 zymogen and cleaved protease domain bands are indicated by *filled* and *open arrowheads*, respectively. *E* and *F*, as controls, HEK293 cells expressing corin were incubated without (−) or with (*+*) monensin (*E*) or BFA (*F*) at 37 °C for 18 h. Corin fragments in cell lysates were analyzed by Western blotting under reducing conditions. Corin zymogen and cleaved protease domain bands are indicated by *filled* and *open arrowheads*, respectively. All experiments were done using an anti-C-terminal V5 tag antibody. Data are representative of at least three experiments. BFA, brefeldin A; HEK293, human embryonic kidney 293 cell line; TMPRSS2, transmembrane protease serine 2.

To understand the subcellular location of TMPRSS2 activation, we treated HEK293 cells expressing the TMPRSS2 WT with trypsin to remove surface proteins and lysed the cells for Western blotting under reducing conditions. We detected the ∼65-kDa zymogen band and the ∼31-kDa protease domain band in the cells with or without trypsin digestion (Fig. 3*A*). In control experiments with corin, a cardiac TTSP that is activated on the cell surface but not intracellularly (39, 47), the ∼40-kDa cleaved protease domain band disappeared after the cells were treated with trypsin (Fig. 3*B*). These results showed that, unlike corin, TMPRSS2 is activated inside the cell.

We then treated the TMPRSS2-expressing HEK293 cells with monensin and brefeldin A (BFA), which inhibit protein trafficking in the Golgi and endoplasmic reticulum (ER), respectively (48). In Western blotting, we observed similar levels of the ∼65- and ∼31-kDa bands in the TMPRSS2-expressing cells without or with monensin treatment (up to 0.9 μM) (Fig. 3*C*). In contrast, BFA treatment reduced the level of the ∼31-kDa protease domain band dose-dependently (Fig. 3*D*). In control experiments with corin, the 40-kDa protease domain band was undetectable in the monensin-treated (Fig. 3*E*) or BFA-treated (Fig. 3*F*) cells, consistent with the extracellular activation of corin (39, 47). These results indicated that TMPRSS2 is activated after exiting the ER, most likely in the Golgi, before reaching the cell surface.

### Inhibition of TMPRSS2 activation cleavage by hepatocyte growth factor activator inhibitors 1 and 2

**Figure 4:**
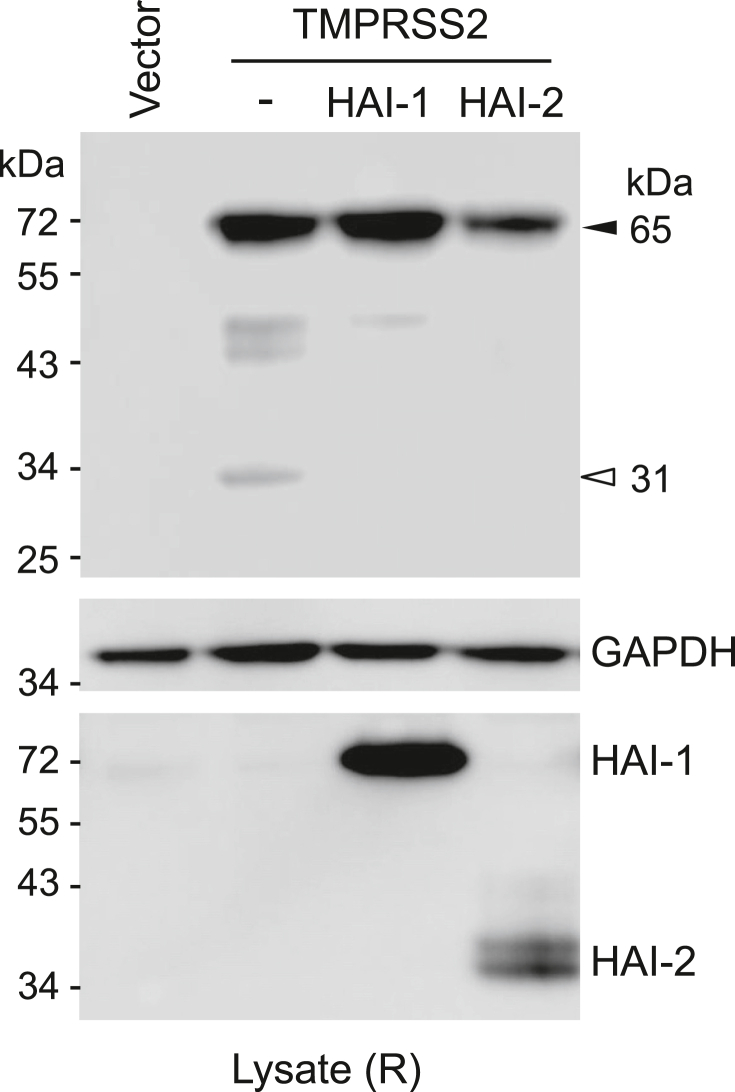
**Inhibition of TMPRSS2 activation cleavage by HAI-1 and HAI-2.** HEK293 cells were transfected with a control vector or a plasmid expressing TMPRSS2 without (−) or with a plasmid expressing HAI-1 or HAI-2. TMPRSS2 fragments in cell lysates were analyzed by Western blotting under reducing conditions (*R*). TMPRSS2 zymogen and protease domain bands are indicated by *filled* and *open arrowheads*, respectively (*top panel*). GAPDH (*middle panel*) as well as HAI-1 and HAI-2 (*bottom panel*) were verified as controls by Western blotting. The data are representative of three experiments. HAI, hepatocyte growth factor activator inhibitor; HEK293, human embryonic kidney 293 cell line; TMPRSS2, transmembrane protease serine 2.

Hepatocyte growth factor activator inhibitors 1 and 2 (HAI-1 and HAI-2) are Kunitz-type inhibitors that inhibit many TTSPs, including TMPRSS2 (49, 50, 51). To verify TMPRSS2 intracellular autoactivation, we coexpressed TMPRSS2 and HAI-1 or HAI-2 in HEK293 cells and analyzed TMPRSS2 activation by Western blotting. We found that the TMPRSS2 activation cleavage was abolished in the HEK293 cells coexpressing HAI-1 or HAI-2, as indicated by the absence of the ∼31-kDa protease domain band (Fig. 4). These results indicated that inhibition of TMPRSS2 activity by HAI-1 or HAI-2 prevents TMPRSS2 intracellular autoactivation in HEK293 cells.

### Effects of N-glycosylation on TMPRSS2 activation

**Figure 5:**
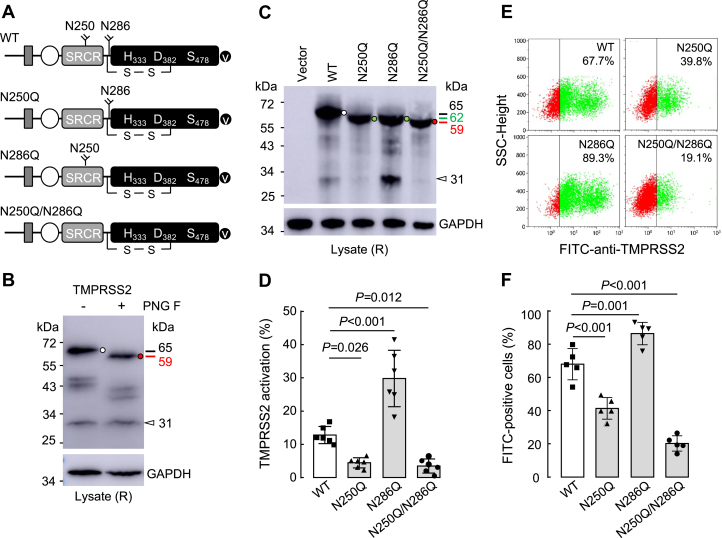
**Analysis of N-glycosylation in TMPRSS2.***A*, illustration of N-glycosylation sites in human TMPRSS2. Asn to Gln substitutions were made at 250 (*N250Q*), 286 (*N286Q*), or 250 and 286 (*N250Q/N286Q*) to abolish the N-glycosylation sites, individually or together. *B*, TMPRSS2 was expressed in HEK293 cells. Cell lysates were treated without (−) or with (*+*) PNGase F (*PNG F*) and analyzed by Western blotting under reducing conditions. The TMPRSS2 zymogen bands at ∼65 kDa (*white dot*) and ∼59 kDa (*red dot*) and the protease domain band (*open arrowhead*) are indicated. Data are representative of three experiments. *C* and *D*, the TMPRSS2 WT and mutants N250Q, N286Q, and N250Q/N286Q expressed in HEK293 cells were analyzed by Western blotting under reducing conditions. The TMPRSS2 zymogen bands at ∼65 kDa (*white dot*), ∼62 kDa (*green dots*), and ∼59 kDa (*red dot*) and the protease domain band at ∼31 kDa (*open arrowhead*) are indicated. Percentages of TMPRSS2 activation cleavage, as measured by the ratios of the protease domain band *versus* the zymogen band intensity, from six experiments were quantified. Data (mean ± SD) were analyzed by one-way ANOVA. *E* and *F*, flow cytometric analysis of the WT and mutants N250Q, N286Q, and N250Q/N286Q on the surface of HEK293 cells. Percentages of TMPRSS2-positive cells are shown (*E*). Quantitative data (mean ± SD) from five experiments were analyzed by one-way ANOVA (*F*). HEK293, human embryonic kidney 293 cell line; PNGase F, peptide-*N*-glycosidase F; TMPRSS2, transmembrane protease serine 2.

N-glycosylation is an important mechanism in the regulation of TTSP folding, intracellular trafficking, and zymogen activation (41, 52, 53). However, the functionality of individual *N*-glycans in a particular protein is hard to predict. There are two predicted N-glycosylation sites in human TMPRSS2, one at N250 in the SRCR domain and other at N286 before the protease domain (Fig. 5*A*). To test if TMPRSS2 is N-glycosylated, we treated lysates from the TMPRSS2-expressing cells with peptide-*N*-glycosidase F (PNGase F) to remove *N*-glycans from glycoproteins. In Western blotting, the ∼65-kDa TMPRSS2 zymogen band migrated faster at ∼59 kDa after the PNGase F treatment, whereas the position of the ∼31-kDa protease domain band remained unchanged (Fig. 5*B*). Similarly, Western blotting of human lung A549 cell lysates showed that endogenous TMPRSS2 migrated faster when the samples were treated with PNGase F (Fig. S4). These results indicated that TMPRSS2 is N-glycosylated in the propeptide region.

To verify these results and understand the effect of N-glycosylation on TMPRSS2 activation, we made mutants N250Q, N286Q, and N250Q/N286Q, in which the N-glycosylation consensus sequences were mutated, individually or together (Fig. 5*A*). We expressed the mutants in HEK293 cells and analyzed them in Western blotting. The zymogen bands from the WT, the mutants N250Q and N286Q, and the mutant N250Q/N286Q migrated at ∼65, ∼62, and ∼59 kDa, respectively (Fig. 5*C*). When the lysates containing the WT and mutants were treated with PNGase F, all the zymogen bands migrated at ∼59 kDa (Fig. S5). These results indicated that both N250 and N286 were N-glycosylated. Intriguingly, the ∼31-kDa protease domain band was barely detectable in the mutants N250Q and N250Q/N286Q but stronger in the mutant N286Q compared with that in the WT (Fig. 5, *C* and *D*). These results indicated that N-glycosylation at N250, but not at N286, is necessary for TMPRSS2 activation in HEK293 cells.

### Effects of N-glycosylation on TMPRSS2 intracellular trafficking

**Figure 6:**
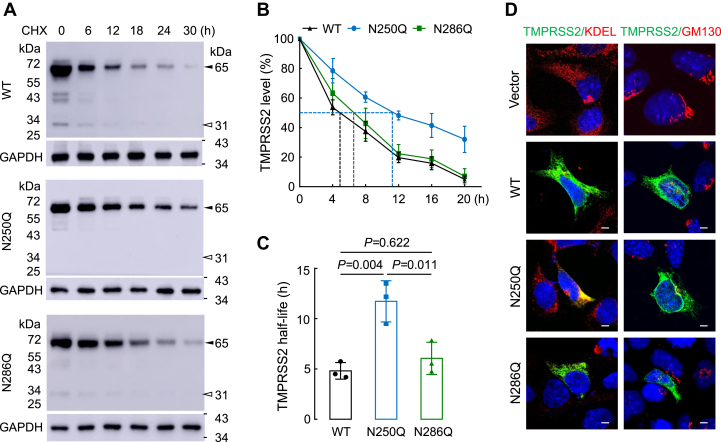
**Analysis of intracellular TMPRSS2 proteins in HEK293 cells.***A*, HEK293 expressing the TMPRSS2 WT and mutants N250Q and N286Q were treated with cycloheximide (CHX) (100 μg/ml) at 37 °C. At indicated time points, the cells were treated with trypsin to remove surface proteins. Cell lysates were prepared and analyzed by Western blotting under reducing conditions. *B* and *C*, levels of TMPRSS2 protein bands on Western blots were quantified by densitometric analysis to calculate protein half-lives. Data are mean ± SD from three experiments. Statistical analysis was done by one-way ANOVA. *D*, coimmunostaining of TMPRSS2 proteins (*green*) with the ER marker KDEL (*red*) (*left column*) or the Golgi marker GM130 (*red*) (*right column*) in HEK293 cells transfected with a vector or plasmids expressing the TMPRSS2 WT and mutants N250Q and N286Q. Cell nuclei were stained with DAPI (*blue*). Data are representative of five experiments. DAPI, 4′,6-diamidino-2-phenylindole; ER, endoplasmic reticulum; HEK293, human embryonic kidney 293 cell line; TMPRSS2, transmembrane protease serine 2.

We next examined the TMPRSS2 WT and mutants N250Q, N286Q, and N250Q/N286Q on the surface of HEK293 cells. In flow cytometry, levels of the mutants N250Q and N250Q/N286Q were lower than those of the WT and mutant N286Q (Fig. 5, *E* and *F*), suggesting that abolishing N-glycosylation at N250, but not N286, inhibited TMPRSS2 trafficking to the cell surface. Consistently, the mutant N250Q had a longer intracellular half-life than those of the WT and mutant N286Q in cycloheximide-based protein chase and Western blotting experiments (11.7 ± 2.1 *versus* 4.8 ± 0.8 h in the WT and 6.1 ± 1.6 h in the mutant N286Q, *p* = 0.004 and 0.011, respectively) (Fig. 6, *A–C*). In immunostaining, strong overlapping staining of the mutant N250Q and the ER marker KDEL was observed in HEK293 cells, whereas such overlapping staining was not observed in WT or the mutant N286Q (Fig. 6*D*, *left column*). In contrast, there was no apparent overlapping staining of the Golgi marker GM130 with the WT or the mutants N250Q and N286Q (Fig. 6*D*, *right column*). Similar overlapping staining with KDEL, but not GM130, was observed in HEK293 cells expressing the mutant N250Q/N286Q (Fig. S6). These results indicated that abolishing N-glycosylation at N250, but not N286, prevents TMPRSS2 from exiting the ER.

### TMPRSS2 N-glycosylation sites in 3D models

**Figure 7:**
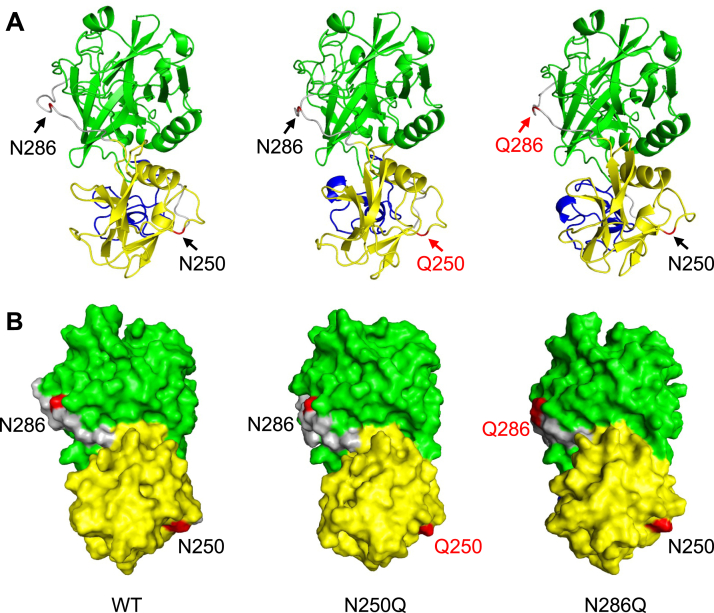
**3D models of TMPRSS2 extracellular modules.** Sequences of the extracellular region of the TMPRSS2 WT and mutants N250Q and N286Q were analyzed by the deep learning RoseTTAFold program. *A*, ribbon models of the TMPRSS2 WT and mutants N250Q and N286Q. The LDLR (*blue*), SRCR (*yellow*), and protease (*green*) modules are displayed. *B*, surface models of the TMPRSS2 WT and mutants N250Q and N286Q. N250, Q250, N286, and Q286 residues are indicated. LDLR, low-density lipoprotein receptor; SRCR, scavenger receptor cysteine-rich domain; TMPRSS2, transmembrane protease serine 2.

Using the artificial intelligence-based RoseTTAFold program (https://robetta.bakerlab.org) for protein structure prediction (54), we built 3D models of the TMPRSS2 extracellular region. The LDLR, SRCR, and protease domains were folded as independent modules (Fig. 7*A*). N250 was in an SRCR domain loop away from the protease domain, whereas N286 was in a linker region near the protease domain. Both N250 and N286 were surface exposed (Fig. 7*B*). Substitution of N250 or N286 with Gln residues did not alter the overall 3D model of the TMPRSS2 extracellular region (Fig. 7, *A* and *B*).

### Interaction of TMPRSS2 with ER chaperones

**Figure 8:**
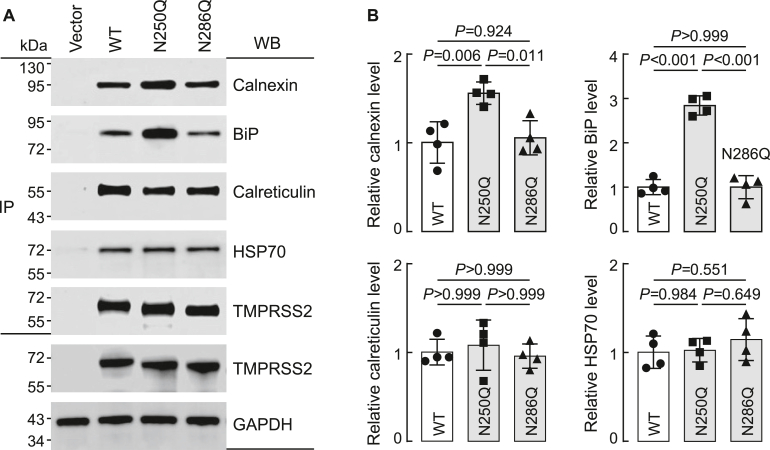
**Analysis of TMPRSS2 and ER chaperone interactions.***A*, lysates were prepared from HEK293 cells expressing the TMPRSS2 WT and mutants N250Q and N286Q. Immunoprecipitation (IP) was conducted using an anti-V5 tag antibody to pull down TMPRSS2 and associated proteins. Then Western blotting (WB) was used to examine ER chaperones calnexin, BiP, calreticulin, and heat shock protein 70 (HSP70) (*rows* 1–4). TMPRSS2 proteins in the pull-down fractions (*row* 5) and in the starting cell lysates (*row* 6) and GAPDH in the starting cell lysates (*bottom row*) were verified, as controls. Data are representative of four experiments. *B*, relative levels of calnexin, BiP, calreticulin, and HSP70 were quantified by densitometric analysis of protein bands on the WBs. Data are mean ± SD from four experiments. *p* Values in calnexin, BiP, and HSP70 analysis were done by one-way ANOVA. *p* Values in calreticulin analysis were done by Kruskal–Wallis test. BiP, binding immunoglobulin protein; ER, endoplasmic reticulum; HEK293, human embryonic kidney 293 cell line; TMPRSS2, transmembrane protease serine 2.

Calnexin binding to *N*-glycans is an important mechanism in glycoprotein folding (55). *N*-glycan elimination prevents client protein folding, causing ER retention of poorly folded proteins *via* direct protein–protein interactions with calnexin and binding immunoglobulin protein (BiP) (52, 56). We immunoprecipitated the WT and mutants N250Q and N286Q in the lysates from HEK293 cells and examined coprecipitated ER chaperones by Western blotting (Fig. 8, *A* and *B*). Levels of calnexin and BiP coprecipitated with the mutant N250Q were higher than those with the WT and mutant N286Q, whereas levels of coprecipitated calreticulin and heat shock protein 70 (HSP70; two other ER chaperones) were comparable among the WT and mutants. In controls, similar levels of the WT and mutants were found in the coprecipitated samples (Fig. 8*A*, row 5) and in the starting cell lysates (Fig. 8*A*, row 6). These results are consistent with the observed ER retention of the mutant N250Q, indicating that N-glycosylation at N250, but not N286, in TMPRSS2 is critical for calnexin-mediated folding in the ER.

### Effects of glucosidase inhibition on TMPRSS2 activation and interaction with calnexin

**Figure 9:**
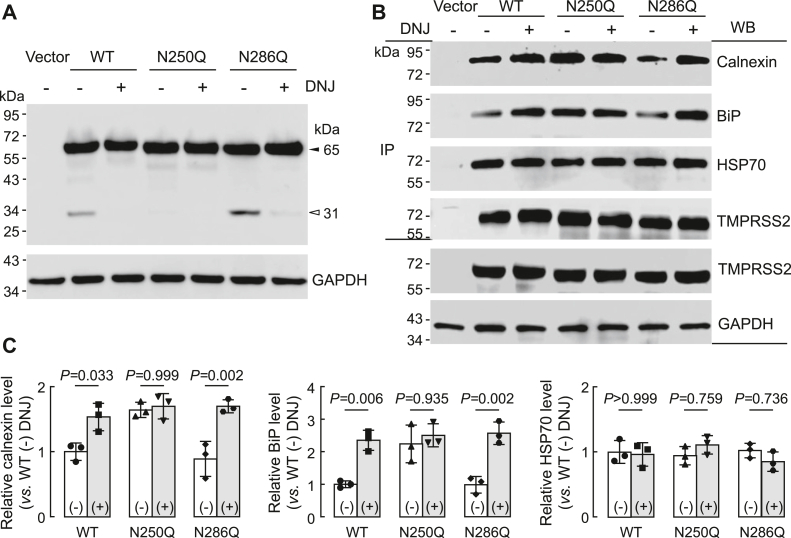
**Effects of DNJ on TMPRSS2 activation and interactions with ER chaperones.***A*, HEK293 cells transfected with a vector or plasmids expressing the TMPRSS2 WT and mutants N250Q and N286Q were treated without (−) or with (*+*) DNJ (2 mM), a glucosidase inhibitor. Cell lysates were prepared. TMPRSS2 fragments were analyzed by Western blotting (WB) using an anti-V5 antibody under reducing conditions. The zymogen band (*black arrowhead*) and the cleaved protease domain band (*open arrowhead*) are indicated. The data are representative of three experiments. *B*, analysis of interactions between TMPRSS2 proteins and ER chaperones in HEK293 cells without (−) or with (*+*) the DNJ treatment. Immunoprecipitation (IP) was done using an anti-V5 antibody to pull down TMPRSS2 and associated proteins. WB was used to examine calnexin, BiP, and HSP70 (rows 1–3). TMPRSS2 proteins in the pull-down fractions (row 4) and in the starting cell lysates (row 5) and GAPDH in the starting cell lysates (*bottom row*) were verified, as controls. Data are representative of three experiments. *C*, relative levels of calnexin, BiP, and HSP70 were quantified by densitometric analysis of protein bands on the WBs. Data are mean ± SD from three experiments. *p* Values were analyzed by one-way ANOVA. BiP, binding immunoglobulin protein; DNJ, 1-deoxynojirimycin; ER, endoplasmic reticulum; HEK293, human embryonic kidney 293 cell line; HSP70, heat shock protein 70; TMPRSS2, transmembrane protease serine 2.

In calnexin-mediated folding, triglucosylated oligosaccharides on glycoproteins must be trimmed by α-glucosidases I and II, consecutively, to monoglucosylated oligosaccharides before the calnexin–*N*-glycan interaction occurs (55). To verify the role of the *N*-glycans on TMPRSS2 in regulating TMPRSS2 activation and binding to ER chaperones, we incubated the HEK293 cells expressing the WT and mutants N250Q and N286Q with 1-deoxynojirimycin (DNJ), an inhibitor of α-glucosidases I and II (57, 58). In Western blotting, we found that the DNJ treatment inhibited TMPRSS2 activation, as indicated by the absence or reduced levels of the ∼31-kDa protease domain band in the WT and mutant N286Q, respectively (Fig. 9*A*). In the coimmunoprecipitation experiment, levels of calnexin and BiP coprecipitated with the WT and mutant N286Q were increased in the DNJ-treated samples, compared with those without the DNJ treatment (Fig. 9, *B* and *C*). In contrast, levels of calnexin and BiP coprecipitated with the mutant N250Q remained high with or without the DNJ treatment (Fig. 9, *B* and *C*). In controls, levels of HSP70 coprecipitated with the WT and mutants N250Q and N286Q were similar with or without the DNJ treatment. Similar levels of the TMPRSS2 WT and mutants were verified in the coprecipitated samples (Fig. 9*B*, row 4) and the starting lysates (Fig. 9*B*, row 5). These results indicated that the inhibition of α-glucosidase activities by DNJ prevented the calnexin binding to the *N*-glycan on the WT and mutant N286Q, which impaired the calnexin-assisted TMPRSS2 folding in the ER, leading to increased direct protein–protein interactions with calnexin and BiP.

### Intracellular cleavage of SARS-CoV-2 S protein by TMPRSS2

**Figure 10:**
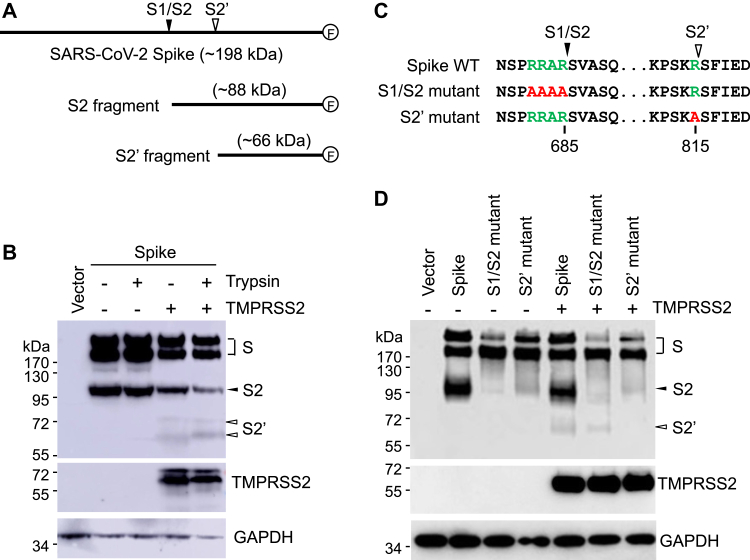
**Intracellular cleavage of SARS-CoV-2 spike protein (S protein) by TMPRSS2.***A*, illustration of SARS-CoV-2 S protein and the fragments cleaved at the S1/S2 and S2′ sites. Molecular masses of the S protein fragments with a C-terminal FLAG tag (*F*) are based on amino acid sequences and predicted N-glycosylation sites. The S protein also has *O*-glycans (79). Actual sizes of the fragments vary depending on experimental settings. *B*, HEK293 cells were transfected with a vector or a plasmid expressing the S protein without (−) or with (*+*) TMPRSS2 coexpression. The cells were treated without (−) or with (*+*) trypsin to remove surface proteins and lysed. The S protein fragments were analyzed by Western blotting using an anti-FLAG antibody under reducing conditions. The S protein and S2 and S2′ fragments are indicated (*top panel*). TMPRSS2 (*middle panel*) and GAPDH (*bottom panel*) in the lysates were verified. *C*, illustration of the S protein cleavage site mutants, in which the S1/S2 and S2′ sites were mutated, respectively. *D*, HEK293 cells transfected with a vector or plasmids expressing the S protein and the S1/S2 and S2′ mutants without (−) or with (*+*) TMPRSS2 coexpression. The S protein fragments were analyzed by Western blotting using an anti-FLAG antibody under reducing conditions (*top panel*). TMPRSS2 (*middle panel*) and GAPDH (*bottom panel*) in the lysates were verified. Data in *B* and *D* are representative of at least three experiments. HEK293, human embryonic kidney 293 cell line; SARS-CoV-2, severe acute respiratory syndrome coronavirus 2; TMPRSS2, transmembrane protease serine 2.

Many studies have shown that furin and TMPRSS2 cleave SARS-CoV-2 S protein at the S1/S2 and S2′ sites, respectively (Fig. 10*A*), although alternative furin and TMPRSS2 cleavages have been reported (20, 21, 59, 60). Given the finding of intracellular TMPRSS2 activation, we tested if TMPRSS2 could cleave SARS-CoV-2 S protein intracellularly. We expressed the full-length SARS-CoV-2 S protein in HEK293 cells and prepared cell lysates before and after trypsin treatment. In Western blotting, the S and S2 fragments of SARS-CoV-2 S protein were detected (Fig. 10*B*, lanes 2 and 3), consistent with the notion that furin, which is abundant in HEK293 cells (61), is primarily responsible for cleaving the S1/S2 site. In HEK293 cells cotransfected with the S protein and TMPRSS2, two additional bands appeared at ∼70 and ∼66 kDa, respectively (Fig. 10*B*, lane 4). These two bands were also observed in the trypsin-treated cells (Fig. 10*B*, lane 5), indicating that they were cleaved by TMPRSS2 inside the cells, consistent with the intracellular TMPRSS2 activation observed in our experiments.

To verify the TMPRSS2 cleavage at the S2′ site, we made S1/S2 and S2′ mutants, in which the furin and TMPRSS2 cleavage sites in the S protein were mutated, respectively (Fig. 10*C*). In Western blotting of lysates from transfected HEK293 cells, the S2 fragment in the S1/S2 mutant was barely detectable without or with TMPRSS2 cotransfection (Fig. 10*D*). In contrast, the S2′ fragment was detected in the cells coexpressing TMPRSS2 and S protein WT and the S1/S2 mutant but not the S2′ mutant (Fig. 10*D*). In controls, similar levels of TMPRSS2 protein were confirmed in the cotransfected cells (Fig. 10*D*). These results indicated that the observed S2′ fragment was derived from TMPRSS2-mediated cleavage at the S2′ site.

## Discussion

TMPRSS2-mediated S protein cleavage is critical in SARS-CoV-2 infection (20, 21). In this study, we examined cellular mechanisms in regulating TMPRSS2 activation and cell surface expression. We expressed the full-length human TMPRSS2, that is, isoform 1, with 529 amino acids, which differs from the originally published human TMPRSS2 isoform 2 with 492 amino acids (1, 62). Compared with TMPRSS2 isoform 2, TMPRSS2 isoform 1 has extra 37 amino acids at the N terminus, probably because of alternative mRNA splicing. To date, there is no evidence indicating that the cytoplasmic extension may alter TMPRSS2 expression or function.

Zymogen activation is a key step in regulating protease activity. In TTSPs, mechanisms underlying zymogen activation vary considerably. For example, corin (39, 47), enteropeptidase (63), and matriptase (40, 64) are activated extracellularly by proprotein convertase subtilisin/kexin 6, trypsin, and prostasin, respectively. Matriptase-2 and hepsin are self-activated on the cell surface (43, 65, 66), whereas TMPRSS11A is self-activated in the ER (67). TMPRSS2 autoactivation has been reported (46); however, the specific subcellular location remains unknown. In this study, we confirmed TMPRSS2 autoactivation in HEK293 cells and human airway 16HBE and lung A549 epithelial cells. Moreover, our data indicated that TMPRSS2 is likely activated in the Golgi, that is, after exiting the ER and before reaching the cell membrane. These results highlight the diversity in zymogen activation mechanisms among TTSPs.

HAI-1 and HAI-2 are inhibitors of many epithelial TTSPs (21, 49). In mice, HAI-1 or HAI-2 deficiency causes embryonic lethality because of dysregulated protease activities (68, 69, 70). Recently, HAI-1 and HAI-2 have been identified as endogenous TMPRSS2 inhibitors in prostate cancer cells (50). In cell-based experiments, HAI-2 inhibits TMPRSS2-mediated influenza A virus and SARS-CoV-2 infection (51, 71). Consistently, cotransfection of HAI-1 or HAI-2 in HEK293 cells blocked TMPRSS2 activation in our study. HAI-1 and HAI-2 are known to act both intracellularly and on the cell surface (44, 72, 73). If HAI-1 and HAI-2 inhibited TMPRSS2 on the cell surface in our experiments, we should still expect to detect the ∼31-kDa protease domain band in cell lysates. In Western blotting, however, we did not observe the ∼31-kDa band, indicating that TMPRSS2 inhibition by HAI-1 and HAI-2 occurred inside the cells. These data are consistent with intracellular TMPRSS2 activation.

N-glycosylation is a key post-translational modification in proteins (74). The consensus N-glycosylation sequence is Asn-X-Ser or Asn-X-Thr, where X can be any amino acid but Pro (75). In human TMPRSS2, there are three Asn-X-Ser sequences at 165 to 167, 250 to 252, and 286 to 288 positions, respectively. However, the sequence at 165 to 167 is Asn-Pro-Ser, which is not an N-glycosylation site. By analyzing the mutants N250Q, N286Q, and N250Q/N286Q, we showed that TMPRSS2 indeed was N-glycosylated at the predicted sites. In TTSPs, the functional importance of the predicted N-glycosylation sites varies extensively. TMPRSS13, for example, has two N-glycosylation sites in its SRCR domain, but those sites are unnecessary for cell surface expression and zymogen activation (41). In contrast, hepsin has one N-glycosylation site in the SRCR domain, which is critical for calnexin-assisted protein folding, ER exiting, cell surface expression, and zymogen activation (53). In this study, we found that the *N*-glycan at N250 in the SRCR domain of TMPRSS2 appears to have a similar role in calnexin-mediated folding and ER exiting. Elimination of the *N*-glycan at this site resulted in increased direct protein–protein interactions with calnexin and BiP, ER retention, and impaired intracellular trafficking, zymogen activation, and cell surface expression. As discussed previously, TMPRSS2 is likely activated in the Golgi. The observed ER retention could explain impaired zymogen activation of the mutant N250Q. Consistently, treatment of DNJ, which inhibits α-glucosidase activities and hence the calnexin–*N*-glycan interaction, also blocked the activation of the TMPRSS2 WT and mutant N286Q in transfected cells.

The phenotypic difference between the mutants N250Q and N286Q is intriguing. Unlike the mutant N250Q, abolishing N-glycosylation at N286 did not impair TMPRSS2 intracellular trafficking, zymogen activation, and cell surface expression. The underlying mechanism is unclear. In our 3D protein modeling, modules in the TMPRSS2 extracellular region appeared to fold independently. The results are consistent with recently published structures of a partial TMPRSS2 extracellular fragment without the LDLR domain (60) and the full-length extracellular region of TMPRSS13 (76). N286 is located between the SRCR and the protease domains. N-glycosylation at this position seems to contribute little to the interaction with calnexin and the folding of the neighboring modules. In support of this hypothesis, studies in blood coagulation factor VII also show that abolishing N-glycosylation in the protease domain, but not between an epidermal growth factor–like and the protease domains, impaired factor VII folding and secretion (77). It is noteworthy that N-glycosylation site at N250 in human TMPRSS2 is conserved in all mammals, whereas the site at N286 is conserved only in primates (Fig. S7), which probably reflects the different functional importance of these two sites observed in our study. Additional studies are required to understand the significance of N-glycosylation at N286 in primate TMPRSS2 proteins.

TMPRSS2 is believed to cleave SARS-CoV-2 S protein at the S2′ site on the cell surface during viral entry, whereas cathepsins cleave the same site intracellularly after clathrin-mediated endocytosis of the viral particles (20). Considering our findings of intracellular TMPRSS2 activation and cleavage of SARS-CoV-2 S protein, it is plausible that TMPRSS2 cleaves SARS-CoV-2 S protein both extracellularly and intracellularly to promote viral entry, replication, and dissemination in host cells. Further studies will be important to verify our findings and to understand the role of TMPRSS2 at various cellular locations during SARS-CoV-2 infection.

In summary, TMPRSS2 is an epithelial TTSP critical in SARS-CoV-2 infection. In this study, we show that TMPRSS2 undergoes intracellular autoactivation and that this process is inhibited by HAI-1 and HAI-2, major inhibitors of epithelial TTSPs. N-glycosylation at an evolutionarily conserved site in the SRCR domain of TMPRSS2 is important for calnexin-assisted protein folding in the ER and subsequent intracellular trafficking, zymogen activation, and cell surface expression. Moreover, we show that TMPRSS2 cleaves SARS-CoV-2 S protein intracellularly in HEK293 cells. Our results provide new insights into the cellular mechanism in regulating TMPRSS2 biosynthesis and function. These findings may also help to elucidate the role of TMPRSS2 in major respiratory viral infection.

## Experimental procedures

### Plasmids

A complementary DNA (cDNA) (NM_001135099.1), encoding human TMPRSS2 (isoform 1), was amplified from human MTC II cDNA panel (Clontech; catalog no.: 636743) and inserted into pcDNA 3.1/V5 plasmid (Thermo Fisher Scientific; catalog no.: K4800-01), as described previously (67). Site-directed mutagenesis (QuikChange Lightning; Agilent Technologies) was used to make plasmids expressing TMPRSS2 mutants N250Q, N286Q, N250Q/N286Q, R292A, and S478A. The amino acid numbering was based on the 529-amino-acid full-length TMPRSS2. All expressed TMPRSS2 proteins had a C-terminal V5 tag. The pCMV3-based plasmid, expressing the 1287-amino-acid full-length SARS-CoV-2 S protein with a C-terminal FLAG tag, was from Sino Biological, Inc (catalog no.: VG40589). Plasmids expressing SARS-CoV-2 S proteins with mutated S1/S2 and S2′ cleavage sites were made by site-directed mutagenesis. Plasmids expressing human corin with a C-terminal V5 tag and human HAI-1 and HAI-2 with an N-terminal FLAG tag were described previously (67, 78).

### Cell lines

HEK293 cells were from the American Type Culture Collection (catalog no.: CRL-1573; short tandem repeat [STR] profiled) and grown in Dulbecco’s modified Eagle’s medium (Corning; catalog no.: 10-013-CVR) with 10% fetal bovine serum (FBS) (Gemini; catalog no.: 900-108). Human bronchial epithelial 16HBE cells (Mingzhoubio; catalog no.: MZ-1420; STR profiled) and human alveolar basal epithelial adenocarcinoma A549 cells (Mingzhoubio; catalog no.: MZ-0015; STR profiled) were grown in minimal essential medium (MEM; Corning; catalog no.: 10-010-CV) and RPMI1640 (Corning; catalog no.: 10-040-CV), respectively, with 10% FBS. Cell culture was kept at 37 °C in humidified incubators with 5% CO_2._

### Transfection and Western blotting

HEK293, A549, and 16HBE cells in 6-well plates (Corning; catalog no.: 3516) were transfected with a control vector and expression plasmids using PolyJet *In Vitro* DNA Transfection Reagent (SignaGen Laboratories; catalog no.: SL100688). After 6 h, the cells were cultured in fresh medium with 10% FBS for 1 to 2 more days, washed with PBS, and lysed with 1% (v/v) Triton X-100, 50 mM Tris–HCl, pH 8.0, 150 mM NaCl, and mixed protease inhibitors (1:100 dilution; Roche Applied Science; catalog no.: 04693116001). Cell lysates with (reducing) or without (nonreducing) 2.5% (v/v) β-mercaptoethanol were analyzed by SDS-PAGE and Western blotting with following antibodies: horseradish peroxidase (HRP)–conjugated anti-V5 (Thermo Fisher Scientific; catalog no.: R96125, 1:5000 dilution), HRP-conjugated anti-FLAG (Sigma; catalog no.: A8592, 1:10,000 dilution), and anti-GAPDH (Bioworld; catalog no.: MB001H, 1:10,000 dilution). To analyze endogenous TMPRSS2 protein in human alveolar A549 cells, an antibody against an epitope in the TMPRSS2 stem region (Abclonal; catalog no.: A1979, 1:500 dilution) was used in Western blotting. Western blots were exposed to chemiluminescent reagents (NCM Biotech; catalog no.: P10050) and analyzed by an imager (Amersham Imager 600; GE Healthcare). Protein bands were quantified by densitometry.

### Peptide substrate assay

A fluorogenic substate assay was used to examine TMPRSS2 catalytic activity. HEK293 cells were grown in 96-well plates and transfected with a vector (control) or plasmids expressing TMPRSS2 proteins. After 24 h at 37 °C, the cells were washed with Opti-MEM (Gibco; catalog no.: 31985050) and incubated with a fluorogenic peptide substrate (Boc-Gln-Ala-Arg-AMC; R&D Systems; catalog no.: ES014) (200 mM in Opti-MEM) at room temperature for 1 h. Fluorescent intensity was monitored in a plate reader (Spectra Max M5; Molecular Devices) with excitation at 380 nm and emission at 460 nm wavelengths.

### Cell surface protein labeling

HEK293 cells expressing TMPRSS2 proteins in 6-well plates were washed with PBS and incubated with sulfo-*N*-hydroxysuccinimide–biotin (0.25 mg/ml, 1 ml/well) (Thermo Fisher Scientific; catalog no.: 89881). After 3 min on ice, a glycine solution (100 mM, 2 ml/well) was added to stop the reaction. After 15 min, the cells were lysed. Biotin-labeled proteins were precipitated with NeutrAvidin beads (Thermo Fisher Scientific; catalog no.: 29201) at 4 °C. After 16 h, the beads were washed with PBS. Proteins were eluted and analyzed by SDS-PAGE and Western blotting.

### Flow cytometry

HEK293 cells transfected with plasmids expressing TMPRSS2 proteins were detached from culture plates with 0.02% (w/v) EDTA and incubated with an anti-V5 antibody (Thermo Fisher Scientific; catalog no.: R96025, 1:500 dilution) followed with an Alexa Fluor 488-labeled secondary antibody (Invitrogen; catalog no.: A21202, 1:500 dilution) or an anti-TMPRSS2 antibody (Abcam; catalog no.: ab280567, 1:200 dilution) followed with an Alexa Fluor 647-labeled secondary antibody (Yeasen; catalog no.: 33113ES60, 1:500 dilution). After washing with PBS, the cells were examined by flow cytometry (Gallios, Beckman Coulter). Pyridinium iodide (Sigma; catalog no.: P8080, 1:1000 dilution) was used for life cell gating. Data were analyzed with Kaluza Analysis Software (Beckman Coulter).

### Immunofluorescent staining

HEK293 cells on glass coverslips were transfected with plasmids expressing TMPRSS2 proteins. After 24 h at 37 °C, the cells were fixed by 4% (v/v) paraformaldehyde (membrane nonpermeable) or cold acetone (membrane permeable) at room temperature for 5 min and treated with 5% (w/v) bovine serum albumin to reduce nonspecific background. Immunostaining was done using anti-V5 (Thermo Fisher Scientific; catalog no.: R96025, 1:500 dilution), anti-TMPRSS2 (Abcam; catalog no.: ab280567, 1:200 dilution), anti-KDEL (ER marker) (Abcam; catalog no.: ab176333, 1:500 dilution), and anti-GM130 (Golgi marker) (Abcam; catalog no.; ab215966, 1:1000 dilution) primary antibodies and Alexa 488- or Alexa 594-labeled secondary antibodies (Thermo Fisher Scientific; catalog nos.: A11008 and A21203, 1:500 dilution). After washing with PBS, a solution with 4′,6-diamidino-2-phenylindole (Southern Biotech; catalog no.; 1000-20) was added to stain cell nuclei. The cells were inspected using a confocal microscope (Olympus FV3000).

### Trypsin treatment of intact cells

To test if TMPRSS2 proteins were on the cell surface, HEK293 cells were transfected with plasmids expressing TMPRSS2 proteins or a control plasmid expressing corin. After 24 h at 37 °C, the cells were treated with 0.25% trypsin–EDTA (w/v) for 1 min. Dulbecco’s modified Eagle’s medium with 10% FBS was added to block trypsin activity. After washing with PBS, the cells were lysed. TMPRSS2 and corin proteins in cell lysates were analyzed by Western blotting using an anti-V5 antibody, as described previously. Similar procedures were used to examine intracellular cleavage of SARS-CoV-2 S protein by TMPRSS2.

### Effects of monensin and BFA

To identify subcellular sites of TMPRSS2 activation, increasing doses of monensin (inhibitor of protein secretory pathway) and BFA (inhibitor of protein trafficking in the ER) were incubated with HEK293 cells expressing TMPRSS2. After 24 h at 37 °C, the cells were lysed, and TMPRSS2 proteins in cell lysates were analyzed by SDS-PAGE and Western blotting.

### Glycosidase treatment

Lysates (20 μg) from A549 cells and transfected HEK293 cells expressing TMPRSS2 proteins were prepared without (nonreducing) or with (reducing) 0.5% SDS and 40 mM dithiothreitol. The samples were heated at 100 °C for 10 min. PNGase F (30 units, purified from *Flavobacterium meningosepticum*, New England Biolabs; catalog no.: P0704S) was added to the lysates and incubated at 37 °C for 3 h. TMPRSS2 proteins in PNGase F-treated samples were analyzed by SDS-PAGE and Western blotting, as described previously.

### Protein chase experiment

HEK293 cells in 6-well plates were transfected with plasmids expressing TMPRSS2 proteins. After 12 h at 37 °C, the cells were treated with cycloheximide (100 μg/ml) (Sigma; catalog no.: C7698) to stop protein synthesis. At specific time points, the cells were treated with trypsin–EDTA (0.25%) at 37 °C for 1 min to remove cell surface proteins and lysed, as described previously. TMPRSS2 proteins in cell lysates were examined by Western blotting.

### Analysis of ER chaperone interactions

To examine ER chaperone interactions, the TMPRSS2 WT and mutants N250Q and N286Q in HEK293 cell lysates were immunoprecipitated with an anti-V5 antibody (Thermo Fisher Scientific; catalog no.: R96025, 1:1000 dilution) and protein A-Sepharose beads (Thermo Fisher Scientific; catalog no.: 101042) to pull down TMPRSS2 and associated proteins. After washing, proteins on the beads were eluted with a sample buffer containing 2.5% (v/v) β-mercaptoethanol and analyzed by SDS-PAGE and Western blotting using following antibodies: anti-V5 (Thermo Fisher Scientific; catalog no.: R96125, 1:5000 dilution), BiP (Cell Signaling; catalog no.: 3177S, 1:1000 dilution), calnexin (Cell Signaling; catalog no.; 2679S, 1:1000 dilution), calreticulin (Cell Signaling; catalog no.: 12238T, 1:1000 dilution), HSP70 (Abcam; catalog no.: ab45133, 1:10,000 dilution), and HRP-conjugated secondary antibodies (Bioworld; catalog no.: BS13278, 1:10,000 dilution).

### Inhibition of glucosidases by DNJ

HEK293 cells expressing the TMPRSS2 WT and mutants N250Q and N286Q in 6-well plates were incubated with or without DNJ (2 mM, Selleckchem; catalog no.: S3839), which inhibits cellular α-glucosidases I and II (57, 58). After 36 h at 37 °C, the cells were washed with PBS and lysed. The lysates were used in Western blotting and coimmunoprecipitation to examine TMPRSS2 activation and interactions with ER chaperones, as described previously.

### Cleavage of SARS-CoV-2 S protein in cotransfected cells

HEK293 cells were transfected with a vector (negative control) or plasmid expressing the full-length SARS-CoV-2 S protein alone or with the plasmid expressing TMPRSS2. After 24 h at 37 °C, the transfected cells were treated with or without trypsin, as described previously, and lysed. To verify the TMPRSS2 cleavage site in the S protein, the S1/S2 and S2′ site mutants were expressed in HEK293 cells with or without TMPRSS2 coexpression. The cells were lysed, and S protein fragments and TMPRSS2 protein in cell lysates were analyzed by SDS-PAGE and Western blotting using HRP-conjugated antibodies against FLAG and V5 tags, respectively. GAPDH on Western blots was verified as a control for protein loading.

### Protein 3D structure modeling

RoseTTAFold software, an artificial intelligence–based deep learning program for protein structure prediction (54), was used to build 3D models to gain insights into TMPRSS2 N-glycosylation sites. The extracellular sequence of TMPRSS2 WT and mutants N250Q and N286Q (Trp143 to the C terminus) was analyzed by RoseTTAFold. The PyMOL program (www.pymol.org) was used to generate 3D ribbon and surface models.

### Statistics

Data were analyzed with Prism 8 software (GraphPad Software, Inc). Normal distribution of the data was verified using Shapiro–Wilk test. If the data passed the test, comparisons among three or more groups were done using one-way ANOVA and Tukey’s post hoc analysis. Otherwise, comparisons were done with Kruskal–Wallis test. Data are presented as mean ± SD. *p* Values of <0.05 were considered significant.

## Data availability

All the data described in this study are presented in the article and accompanying supporting information.

## Supporting information

This article contains supporting information.

## Conflict of interest

The authors declare that they have no conflicts of interest with the contents of this article.
