# Supplementary material for: Transmembrane serine protease TMPRSS2 implicated in SARS-CoV-2 infection is autoactivated intracellularly and requires N-glycosylation for regulation
Source: J Biol Chem. 2022 Oct 26;298(12):102643. doi: 10.1016/j.jbc.2022.102643 (PMC9598255; doi:10.1016/j.jbc.2022.102643)
Supplement: Supporting information [file mmc1.pdf]

Supporting Information

Transmembrane serine protease TMPRSS2 implicated in SARS-CoV-2 infection is autoactivated intracellularly and requires N-glycosylation for regulation

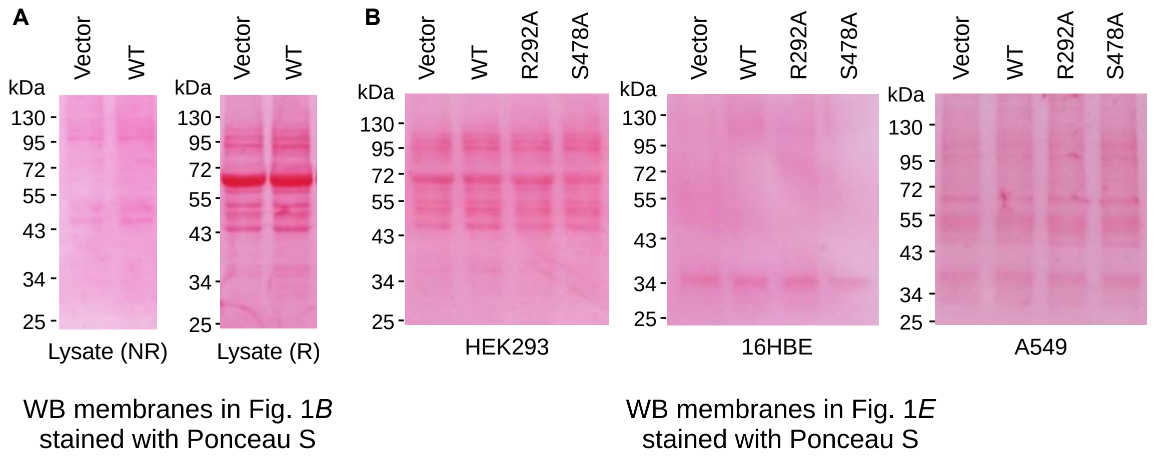

Figure S1. Ponceau S-stained western blot membranes. *A* and *B*, In addition to the GAPDH controls shown in Fig. 1, *B* and *E*, the western blot membranes used in those experiments, *i.e.*, Fig. 1, *B* (A) and *E* (B) were stained with ponceau S, as another control for protein sample loading. Comparable levels of non-specific bands were observed in individual lanes in each blot.

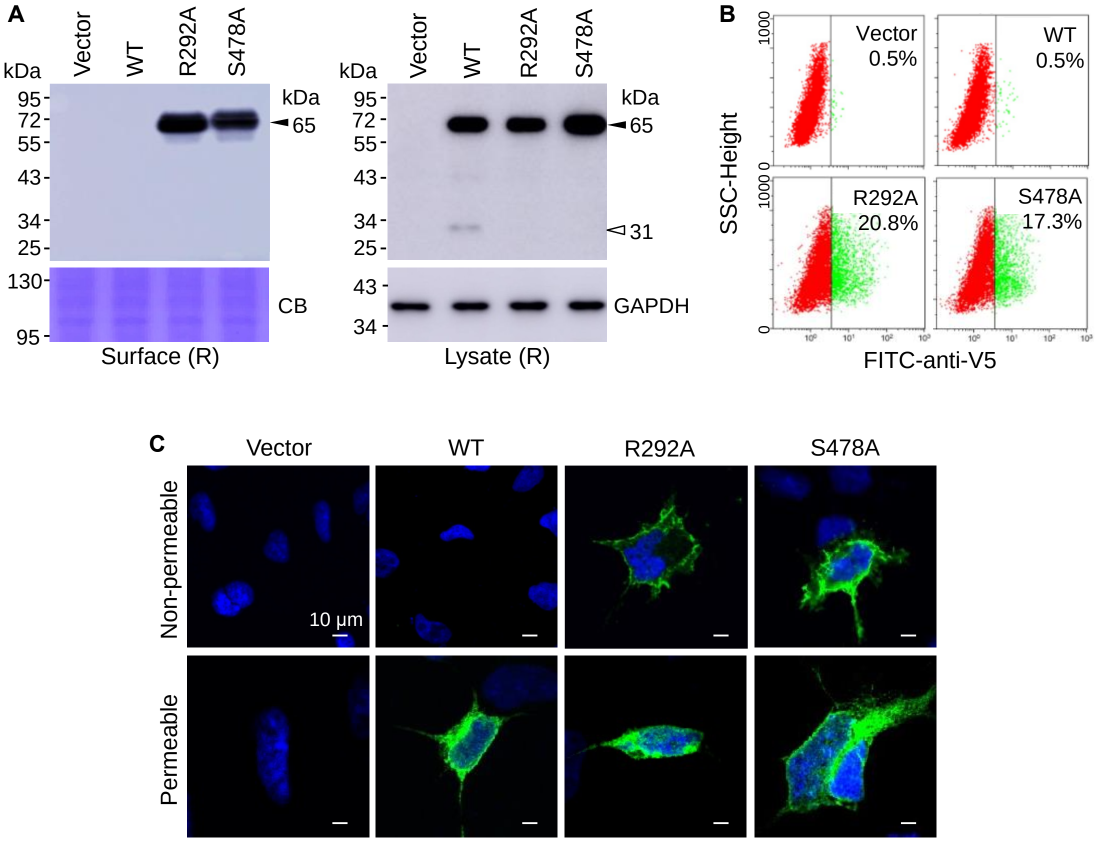

Figure S2. Analysis of cell surface TMPRSS2 proteins using an anti-V5 antibody. *A*, Western blotting of the TMPRSS2 WT and the mutants R292A and S478A in biotin-labeled membrane protein fractions (*left*) and in lysates (*right*) from transfected HEK293 cells. The TMPRSS2 zymogen and cleaved protease domain bands are indicated by black and open arrowheads, respectively. The blotting was done under reducing (*R*) conditions. Coomassie blue (*CB*)-stained non-specific bands (*left*) and GAPDH protein (*right*) were included as protein loading controls. *B*, Flow cytometric analysis of TMPRSS2 proteins on the surface of HEK293 cells transfected with a vector or plasmids expressing the TMPRSS2 WT and the mutants R292A and S478A. Percentages of TMPRSS2-positive cells are indicated. *C*, Immunostaining of TMPRSS2 proteins (*green*) in HEK293 cells transfected with a vector or plasmids expressing the TMPRSS2 WT and the mutants R292A and S478A. Experiments were done under cell membrane non-permeabilized (*top row*) and permeabilized (*bottom row*) conditions. Cell nuclei were stained by DAPI (*blue*). Scale bars: 10 µm. All experiments were done using an anti-C-terminal V5 tag antiboy. Data are representative of at least three experiments.

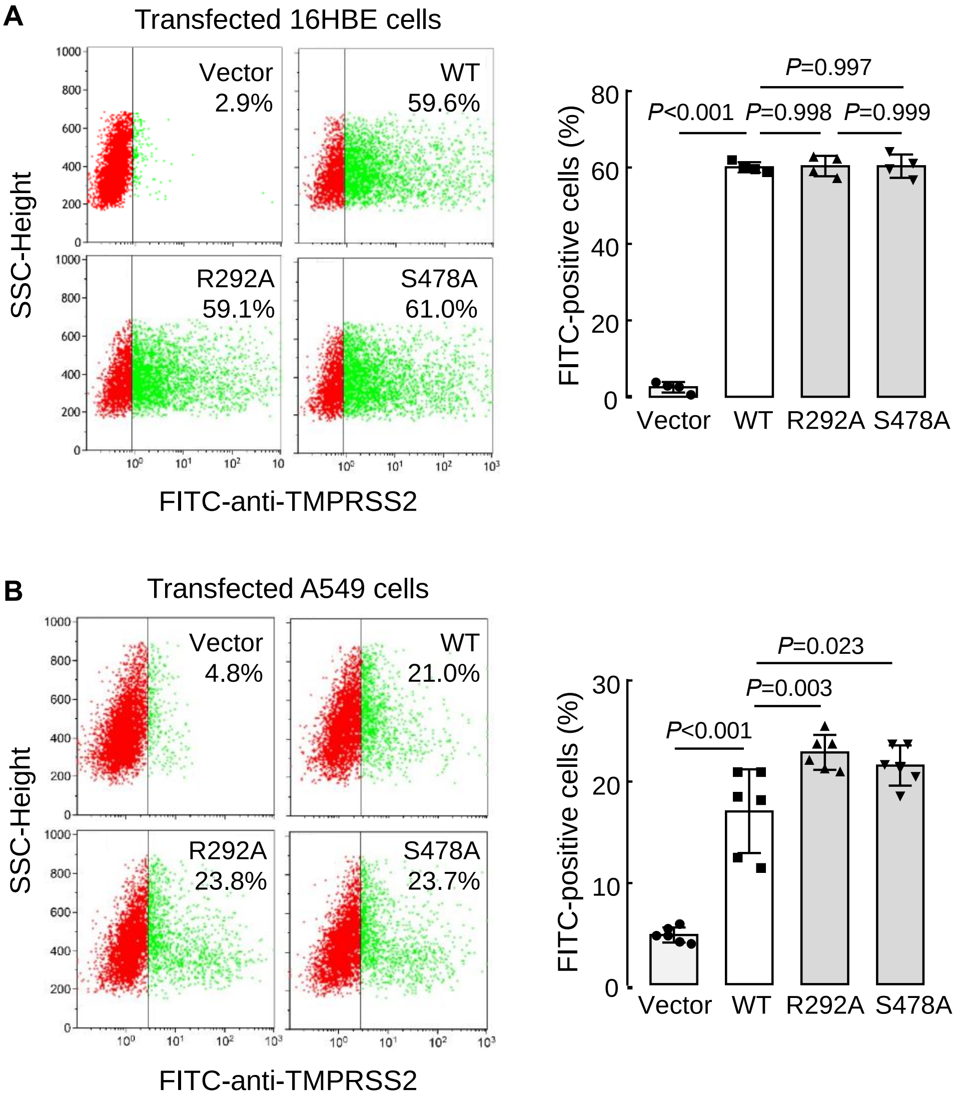

Figure S3. Expression of TMPRSS2 proteins on human airway and lung epithelial cell surface. *A* and *B*, Human bronchial epithelial 16HBE cells (*A*) and lung epithelial A549 cells (*B*) were transfected with a control vector or plasmids expressing the TMPRSS2 WT and the mutants R292A and S478A. TMPRSS2 proteins on the cell surface were examined by flow cytometry using an anti-TMPRSS2 antibody. Percentages of TMPRSS2-positive cells are indicated. Quantitative data (mean ± SD) from four (*A*) and six (*B*) experiments were analyzed by one-way ANOVA.

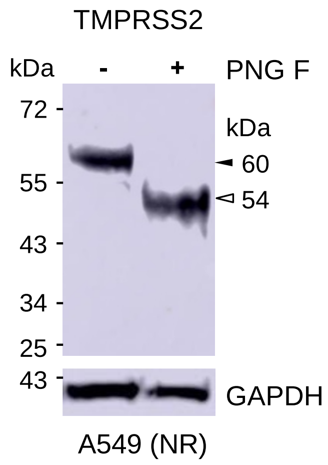

Figure S4. Analysis of N-glycosylation in endogenous TMPRSS2 in human A549 cells. Lysates from human lung epithelial A549 cells were prepared and treated without (-) or with (+) PNGase F (*PNG F*) at 37ºC for 3 h. Endogenous TMPRSS2 was analyzed by western blotting under non-reducing (*NR*) conditions using an antibody against an epitope in the extracellular stem region of human TMPRSS2. The TMPRSS2 bands at ~60 kDa and ~54 kDa are indicated by black and open arrowheads, respectively. GAPDH was used as a control.

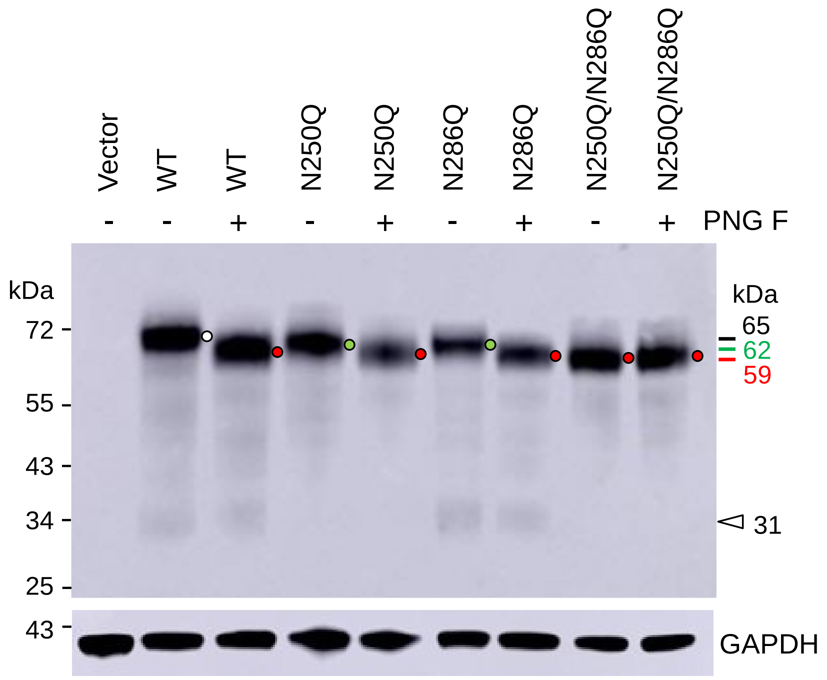

Figure S5. Analysis of N-glycosylation in the TMPRSS2 WT and mutants. HEK293 cells were transfected with a vector or plasmids expressing the TMPRSS2 WT and the mutants N250Q, N286Q, and N250Q/N286Q. The cells were lysed and the lysates were treated without (-) or with (+) PNGase F (*PNG F*). TMPRSS2 proteins were analyzed by western blotting under reducing conditions using an anti-V5 antibody. The TMPRSS2 zymogen bands at ~65 kDa (*white dot*), ~62 kDa (green dots), and ~59 kDa (*red dot*) and the protease domain band (*open arrowhead*) are indicated. Data are representative of three experiments.

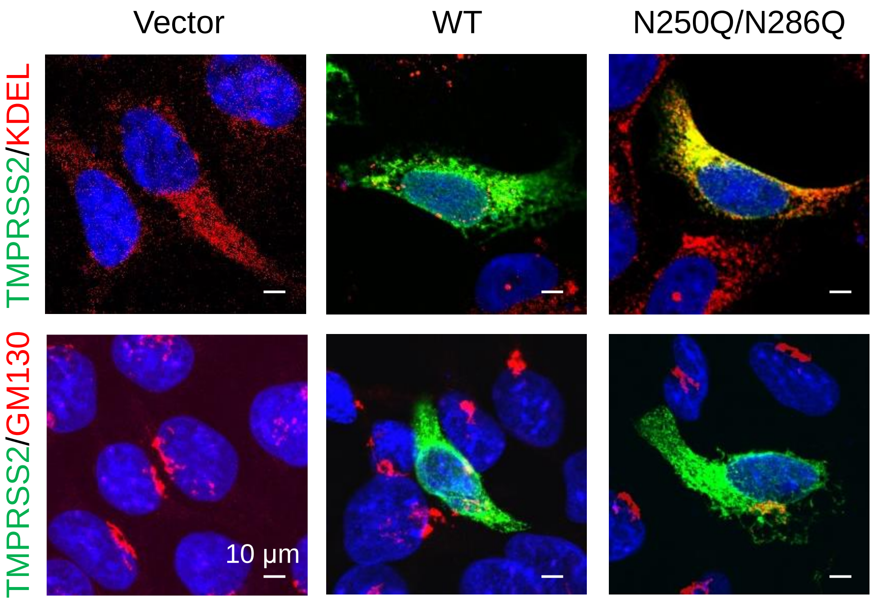

Figure S6. Immunostaining of TMPRSS2 proteins in transfected HEK293 cell. The TMPRSS2 WT and the mutant N250Q/N286Q were expressed in HEK293 cells. Co-immunostaining of TMPRSS2 proteins (*green*) with the ER marker KDEL (*red*) (*top row*) or the Golgi marker GM130 (*red*) (*bottom row*) was done in HEK293 cells transfected with a vector or plasmids expressing the TMPRSS2 WT and the mutant N250Q/N286Q. Cell nuclei were stained by DAPI (*blue*). Data are representative of three experiments.

	250 … 286
**Primates**	
Human	klntsagnvdiykklyhsdacsskavvslrciacgvnlnssrq
Chimpanzee	klntsagnvdiykklyhsdacsskavvslrciacgvnlnssrq
Gorilla	klntsagnvdiykklyhsdacsskavvslrciacgvnlnssrq
Gibbon	klntsarnvdiykklyhsdacsskavvslrciacgvnlnssrq
Rhesus monkey	klntsagnvdiykklyhsdacsskavvslrciacgvrsnlsrq
Lemur	klntsassvdiykklyhsascsskavvslrciacgttsnvssq
Marmoset	klnlsagnvdiykklyhsgtcsskavvslrcvacgvtlnssrq
**Non-primates**	
Cattle	klnisandidlykklyhsdvcssktvvslrciecgvsvktsrq
Horse	klntsadnidlykklyhsdvcsskkvvsl-cmecgvsskqqsr
Pig	klnksannmdlykklyhsdvctsntvvslrciecgvsgkmsnr
Dog	klnisaghmdlykklyhsdvcssktvvslrciecgvsakgsrq
Tiger	rvnasahhvdlykklyhsdvcssktvvslrciecgvtakmgrq
Cat	rvntsanhmdlykklyhsdvcssktvvslrciecgvtakmgrq
Rat	klnvsagnvdlykklyhsdscssrmvvslrciecgvrsvrrqs
Mouse	klnvssgnvdlykklyhsdscssrmvvslrciecgvrsvkrqs
Bat	klnlsashsdlyqklyhsdvcssktvvslrciecgvnrkmgrq

Figure S7. Alignments of N-glycosylation sites in mammalian TMPRSS2 proteins. Partial TMPRSS2 sequences of selected primate (*top*) and non-primate mammalian (*bottom*) species from the NCBI protein database (www.ncbi.nlm.nih.gov/protein) are aligned. Position numbers are based on the full-lengh human TMPRSS2 with 529 amino acids. N-glycosylation sites are in red. Cysteine residues conserved amone all the species are in green.
